# CRISPR technologies and the search for the PAM-free nuclease

**DOI:** 10.1038/s41467-020-20633-y

**Published:** 2021-01-22

**Authors:** Daphne Collias, Chase L. Beisel

**Affiliations:** 1 Department of Chemical & Biomolecular Engineering, North Carolina State University, Raleigh, NC 27695-7905 USA; 2 Helmholtz Institute for RNA-based Infection Research (HIRI)/Helmholtz Centre for Infection Research (HZI), 97080 Würzburg, Germany; 3 Medical Faculty, University of Würzburg, 97080 Würzburg, Germany

**Keywords:** Synthetic biology, CRISPR-Cas9 genome editing

## Abstract

The ever-expanding set of CRISPR technologies and their programmable RNA-guided nucleases exhibit remarkable flexibility in DNA targeting. However, this flexibility comes with an ever-present constraint: the requirement for a protospacer adjacent motif (PAM) flanking each target. While PAMs play an essential role in self/nonself discrimination by CRISPR-Cas immune systems, this constraint has launched a far-reaching expedition for nucleases with relaxed PAM requirements. Here, we review ongoing efforts toward realizing PAM-free nucleases through natural ortholog mining and protein engineering. We also address potential consequences of fully eliminating PAM recognition and instead propose an alternative nuclease repertoire covering all possible PAM sequences.

## Abstract (web summary)

One of the key limitations of CRISPR-Cas-based genome editing techniques is the PAM dependency. Here, the authors review ongoing efforts towards realizing PAM-free nucleases, address potential consequences of eliminating PAM recognition, and propose an alternative nuclease repertoire covering all possible PAM sequences.

## Introduction

The world of biotechnology has undergone a seismic shift with the arrival of CRISPR technologies. These technologies rely on a CRISPR-associated (Cas) nuclease paired with a guide RNA (gRNA). The ~20–30-nt guide portion of the gRNA helps the nuclease find complementary nucleic-acid sequences, and the nuclease enzymatically cleaves these sequences. This programmable and sequence-specific capability has improved existing approaches or catalyzed the development of new approaches that have collectively led to the shift. As one example, genome editing can be performed by cleaving specific DNA sequences and guiding the repair process, whether for reversing genetic diseases, improving traits of crop plants, or studying the genetic basis of cellular functions. In addition, gene expression can be selectively activated or repressed at an individual or multiple loci to tune the level of gene expression and alter cellular behavior^1,2^. CRISPR has also been used for a growing class of in vitro diagnostics that rapidly screen for specific nucleic acid sequences in a patient sample with single-base resolution^3^. Many other applications of CRISPR technologies also exist, such as high-throughput screens, gene drives, tailored-spectrum antimicrobials, recorders of transcriptional profiles and cellular fate, and more^4^.

**Fig. 1:**
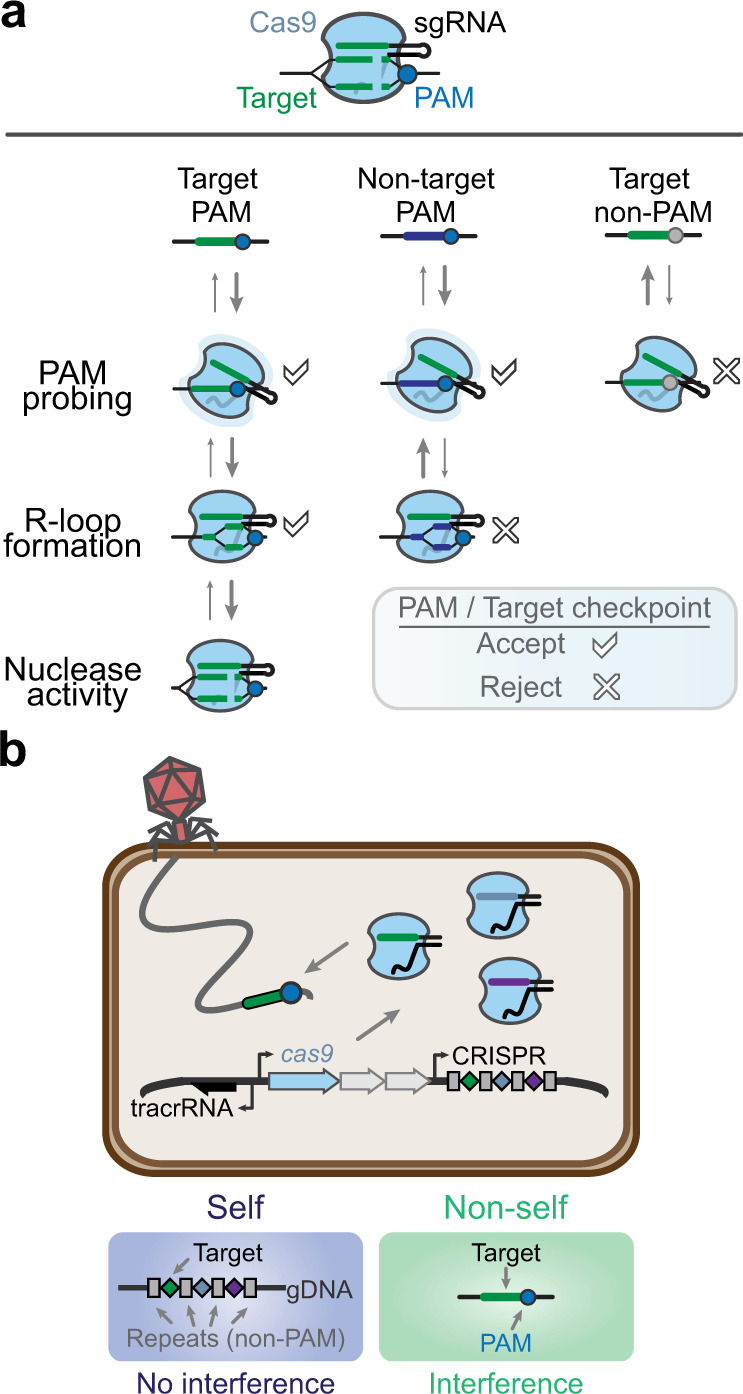
The PAM in target recognition and self/nonself-discrimination for CRISPR–Cas systems. **a** Two checkpoints, the protospacer adjacent motif (PAM) and a flanking target matching the guide, for successful recognition by a Cas nuclease. Cas9 and a single-guide RNA (sgRNA) are used as a representative example. A matching PAM and target results in R-loop formation and target cleavage, whereas either a non-PAM or a nonmatching target block either recognition step by the Cas nuclease. **b** Role of the PAM in self- versus nonself-recognition in prokaryotic immune defense. Self refers to each spacer within the CRISPR array encoded on the host’s genome or endogenous plasmids, whereas nonself refers to invading nucleic acids such as phage or exogenous plasmids. The sgRNA has been engineered for ease-of-use as a fusion between the processed tracrRNA and CRISPR RNA molecules found in the native system.

The ever-expanding list of applications has come with a push to improve the overall utility and flexibility of CRISPR technologies. One restrictive barrier has been the targetable sequences for a given Cas nuclease. Successful targeting requires two factors: extensive complementarity between the gRNA guide and the nucleic acid target, and a short sequence flanking the target typically called a protospacer-adjacent motif (PAM) (Fig. 1a). While some factors influence which sequences can be selected as targets (e.g., the presence of similar off-target genomic sites, GC content, and internal secondary structure)^5^, generally a guide can be created for any target. The PAM requirement, however, is far less flexible (see Box 1). The nuclease scans available DNA for a PAM before probing guide-target complementarity (Fig. 1a). Consequently, a sequence with perfect complementarity to the guide but lacking a PAM will be ignored by the nuclease. The PAM requirement, therefore, serves as a gatekeeper for targeting by CRISPR–Cas.

While a limitation to target selection, the PAM plays an essential role in the natural function of CRISPR–Cas systems, the source of CRISPR technologies (Box 1). The PAM allows these prokaryotic immune systems to differentiate between the DNA target in foreign genetic material (nonself) and the same DNA sequence encoded within CRISPR arrays (self) that produce the RNA guides (Fig. 1b). Without the PAM requirement, CRISPR–Cas systems would target their CRISPR arrays, leading to a potentially catastrophic autoimmune response. Virtually all CRISPR nucleases require a PAM in one form or another. However, the recognized PAM sequences are not shared by all Cas nucleases and instead vary widely, with different sequences, lengths, complexities, orientations, and distances from the target (Supplementary Data 1)^6–50^. This requirement restricts our ability to target any sequence with CRISPR and has led to widespread efforts to relax the PAM requirement, even to the point that nearly any sequence would be recognized as a PAM.

Here, we review efforts to-date that have involved mining natural orthologs and engineering a few well-characterized nucleases for relaxed or altered PAM requirements. We also explore the ramifications of achieving a truly PAM-free nuclease and propose a competing approach based on assembling a repertoire of PAM-dependent nucleases that collectively recognize all possible sequences. PAM determination methods have also been critical to elucidate sequences recognized by each nuclease (Box 2) and have been reviewed previously^51^. Overall, this review addresses a rapidly developing sector of CRISPR technologies that could redefine our ability to target any sequence at will.

Box 1. PAM origins and mechanisms As part of target recognition, Cas nucleases proceed through two checkpoints. First, the nuclease assesses the sequence flanking the intended target (Fig. 1a). For DNA-targeting nucleases, this sequence is often one or multiple sequences collectively called a protospacer adjacent motif, or PAM^51,60^. In contrast, RNA-targeting nucleases (e.g., type III Csm/Cmr complex, type VI Cas13) have been shown to evaluate complementarity between the flanking sequence and a handle sequence encoded within the gRNA^69^. As the second checkpoint, the nuclease assesses base pairing between the guide and the DNA target strand through R-loop formation (Fig. 1a). If both checkpoints are passed, then the nuclease cleaves the target through its specific mechanism-of-action. The PAM, therefore, serves as an essential gatekeeper preventing the nuclease from accessing certain DNA sequences, even if they harbor complete complementarity to the guide.

The gatekeeper function of the PAM is rooted in the natural source of CRISPR technologies and Cas nucleases: CRISPR–Cas systems. These adaptive immune systems native to bacteria and archaea encode their gRNAs within unique patterns of DNA called CRISPR arrays. The arrays comprise alternating conserved repeats and guide-encoding spacers, with each spacer acquired from a previously encountered bacteriophage or another mobile genetic element. By storing the invader-derived sequence that gives rise to a gRNA, CRISPR–Cas systems inherently face a potentially fatal predicament: the DNA encoding the guide would also yield extensive complementarity to the guide. Thus, there lies the potential for each spacer to be recognized as the original invader, leading to genome attack. However, the flanking repeat lacks the PAM recognized by the Cas nuclease (Fig. 1b), allowing the nuclease to effectively ignore this ever-present opportunity for autoimmunity. The PAM, therefore, allows the nuclease to discriminate between subsequent infection by the invader (nonself) from the invader-derived spacer sequence encoded in the CRISPR array (self). Accordingly, CRISPR–Cas systems would be under stringent selective pressure to evolve and maintain PAM recognition as an absolute requirement of immune function. Fortunately for PAM engineering, the presence of this selective pressure also implies that PAM recognition could be undone outside of the natural context of Cas nucleases.

The molecular details of PAM recognition have been revealed for some canonical Cas nucleases. Cas9 from *Streptococcus pyogenes* (SpyCas9) has been characterized the most extensively, where structural analyses and subsequent biochemical assays revealed a series of steps that drive PAM recognition^105^. Briefly, two arginines within the PAM-interacting domain (PID) recognize adjacent guanines on the nontarget strand of the NGG consensus PAM. Recognition is further stabilized by nonspecific interactions with DNA adjacent to the PAM^106^. Residues Ser1109 and Glu1108 within the PID form a phosphate lock with the phosphate on the target strand linking the N nucleotide of the PAM and the first nucleotide of the target sequence complementary to the guide. These events release binding energy that initiates strand separation and R-loop formation.

Characterization of PAM recognition by other Cas nucleases revealed variations on this theme. For example, Cas9 nucleases that are phylogenetically distinct from SpyCas9 rely on a phosphate lock to drive R-loop formation, but read out their consensus PAM using residues within the PI and WED domains^48,49,107,108^. Some of these Cas9 nucleases also recognize specific bases on both DNA strands^48,108^, while molecular-modeling efforts have suggested contributions from van der Waals interactions^108^. Separately, Cas12a nucleases rely on three distinct domains (PI, REC1, and WED) to recognize the PAM. Recognition occurs not only through detecting specific bases but also the shape of the double-stranded DNA and actively rejecting non-PAM sequences. A separate interaction also occurs with the phosphate separating the PAM and target, akin to the phosphate lock for Cas9^109^. Finally, the multiprotein subunit effector complex from Type I CRISPR–Cas systems relies on the recognition of specific bases and DNA shape within the major or minor groove of the PAM DNA. The characterized Cascade complexes also drive a protein wedge into the DNA to force the two strands apart and promote R-loop formation^110^, in contrast to the phosphate lock exhibited by Cas9 and Cas12a. More details concerning the location and composition of the PAM as well as the molecular mechanisms of PAM recognition can be found in multiple recent reviews^51,110,111^. Overall, existing molecular insights into PAM recognition have inspired how PAM recognition could be altered—or even relieved.

Box 2. PAM determination methods Elucidating the set of recognized PAM sequences has been a key step when mining natural Cas orthologs or engineering PAM recognition. As a result, a variety of determination methods have been developed and implemented. Each method can be generally classified based on the use of bioinformatics or experimental approaches or the use of experimental approaches further divided based on whether the assay is in vitro or in vivo and relies on target binding or cleavage. Bioinformatics methods align CRISPR spacers from a nuclease’s natural CRISPR–Cas system to matching sequences (e.g., in plasmids and bacteriophages) in available databases, with the flanking sequence representing a PAM. The drawbacks to this approach are that flanking sequences are specific to the acquisition rather than the nuclease, few (if any) matching sequences are often identified, and the flanking sequences could have been mutated as part of CRISPR avoidance. Instead, experimental methods based on next-generation sequencing (NGS) are commonly employed to elucidate the nuclease’s recognized PAMs. In vitro methods typically involve subjecting a library of potential PAM sequences to NGS after cleavage by purified nuclease with a gRNA^24,26^ or by whole-cell lysate with expressed nuclease and an added gRNA transcribed in vitro^21,76^. An adapter sequence is ligated onto the cleaved sequences to enrich recognized PAM sequences^21,24,26^. Alternatively, PAMs can be determined by the extent of depletion compared to the original library or a nontargeted control^50,76^. Aside from assaying for DNA cleavage events, base-editing events can also be evaluated in vitro to evaluate the depleted PAM preference of edited sequences from a cytosine base editor^76^. In vivo methods based on target cleavage have relied on three approaches: clearance of a plasmid encoding the target and PAM library in bacteria^22^, selecting gRNAs that target along the genome of an infecting RNA phage in bacteria^112^ or evaluating editing frequencies using constructs encoding both the guide RNA and target in human cells^113^. Separately, two different methods in bacteria link target binding by a catalytically dead nuclease to green fluorescent protein fluorescence or growth that both enrich for recognized PAM sequences^25,46^. Finally, cell-free transcription–translation systems (TXTL) offer a more rapid and scalable means to determine PAM sequences by eliminating cell transformation and growth as well as protein and RNA purification^50^.

PAM recognition by a nuclease is a biophysical process that should remain the same whether operating in vitro, in TXTL, in bacteria, or in human cells. However, each of the available PAM determination methods has distinct properties that can yield differences in the elucidated PAMs. For example, binding appears to be more promiscuous than cleavage^89^, while DNA cleavage does not necessarily yield a detectable edit. Separately, higher concentrations of nuclease–gRNA complexes can boost the recognition of less-preferred PAMs, as shown by varying the concentration as part of an in vitro DNA cleavage assay^24^. While the consensus PAM is not expected to change, less-preferred PAMs can be given greater weight or be present or absent depending on the selected method. As general guidance, we recommend noting the method used to elucidate a given PAM as well as the selected conditions. When relying on the elucidated PAM for a given application, the PAM would be more reliable if the determination method closely parallels the application (e.g., methods based on target binding for applications in gene regulation).

There are also different approaches to convey the output of high-throughput PAM determination methods that trade-off simplicity and information content. A consensus sequence or motif (e.g., NGG for SpyCas9) represents the simplest approach, which facilitates the search for potential target sites. However, relying on a single motif often leaves out less-preferred sequences. Sequence logos convey nucleotide bias within a given position, capturing some bases that would not be present in a consensus motif. However, extracting individual sequences and their extent of recognition as PAMs is difficult, given the lack of individual sequences and their recognition by the nuclease. Finally, PAM wheels capture the full diversity of sequences and their relative recognition as PAMs, although extracting a single-consensus sequence or motif is more challenging with PAM wheels than with sequence logos^46^. These different means of conveying PAMs are discussed in detail in prior reviews^51^. Nevertheless, the method of conveying a PAM preference is important to mention here as it can impact how we understand the nuclease’s targeting requirements and therefore their application in downstream technologies.

## A growing need for flexible targeting with Cas nucleases

The need for relaxed PAM requirements did not immediately emerge from the first use of CRISPR technologies; instead, the need developed as the technologies advanced and expanded. The first CRISPR technology was used to introduce insertions or deletions (indels) through nonhomologous end joining that was intended to disrupt the functional expression of a gene^52,53^. Disruptive indels could be introduced in many locations within a gene, placing few restrictions on potential targets. However, rules governing on-target activity or the propensity for off-targeting eliminate certain potential targets from consideration^54^. Separately, dual nucleases have been used in different applications such as dual nicking with reduced off-target effects^55^, where targeting activity is intimately dependent on the orientation and spacing of the two DNA targets. Some technologies are even more restrictive by requiring that a specific location be targeted, such as when introducing defined edits via homologous recombination or prime editing^56^, activating gene expression in bacteria^57^, or detecting single-nucleotide polymorphisms as part of in vitro diagnostics^3^. A poignant example involves base editors, which rely on a DNA-modifying domain that acts on a specific stretch of the target. The positioning of this editing window is principally determined by the PAM; possessing flexibility in the PAM is absolutely crucial given that the window can be as small as one or two nucleotides^58^ or has to be precisely positioned to avoid editing adjacent bases. Therefore, there has been a more recent yet concerted push to expand recognized PAMs to accommodate the growing suite of CRISPR technologies. The push has come in two general forms: mining the natural world for new orthologs of Cas nucleases and employing protein engineering to alter PAM recognition by well-characterized nucleases.

## Mining natural Cas orthologs for altered PAM recognition

Early efforts to co-opt Cas nucleases as technologies gave little consideration for the PAM, although these efforts hinted at the natural diversity of PAM recognition (Supplementary Data 1). In those days, multiple Cas9 nucleases from model bacteria were being characterized, with an eye toward harnessing these nucleases for some level of editing in different cellular contexts or finding active variants that could be packaged into viral delivery vectors^20,21,59^. The Cas9 from the human pathogen *Streptococcus pyogenes* (SpyCas9) immediately jumped to the forefront, in part because of its simple NGG PAM (N = any base). At the same time, the characterization of other Cas9 nucleases revealed entirely distinct consensus PAMs. These other Cas9 nucleases included one from the CRISPR1 locus (Sth1Cas9) in the model lactic-acid bacterium *Streptococcus thermophilus* recognizing an NNATAAW (W = A, T) consensus PAM. The Cas9 from the pathogen *Staphylococcus aureus* (SauCas9), initially lauded for being shorter than SpyCas9 by 315 amino acids, recognizes an NNGRRT consensus PAM (R = A, G). The Cas9 from pathogen *Neisseria meningitidis* (NmeCas9) reflected a larger extreme with an NNNNGATT consensus PAM. These few examples hinted at the natural diversity of Cas9 nucleases.

The consensus motifs of the original Cas9 nucleases were primarily derived from analyzing phage sequences targeted by CRISPR spacers^60^, which are skewed toward sequences recognized through adaptation rather than interference. In contrast, measuring DNA target binding or cleavage has offered a direct readout of PAM preferences (Box 2). Related efforts have revealed more flexibility than that of a simple consensus. For instance, the first high-throughput screen for PAMs recognized by SpyCas9 based on plasmid clearance in *Escherichia coli* identified NAG as a PAM, albeit with weaker recognition than NGG^22,23^. Subsequent work from multiple groups has shown that SpyCas9 can also weakly recognize NGA, NNGG, and a selection of other sequences^21–25^, reflecting a general preference for purines as well as some flexibility in the PAM gap—the distance between the target and first, defined base. While recognition can come from excess nuclease concentrations that can be readily avoided^24^, many of these sequences were identified and validated under setups reflecting practical applications of CRISPR technologies, such as plasmid clearance in bacteria, DNA binding for gene regulation, or indel formation in mammalian cells^22–25^. High-throughput screening indicated that virtually all of the originally characterized Cas9 nucleases also recognize less-preferred PAMs^20–23,25^, representing a common theme for CRISPR nucleases. These studies underscore that PAMs are not solely a consensus sequence or a motif and instead represent a landscape of sequences with different extents of recognition. Furthermore, these studies have led to less-preferred PAMs being factored into off-target predictions^61–63^ and serving as a starting point for boosting recognition of less-preferred sequences as part of PAM engineering.

**Fig. 2:**
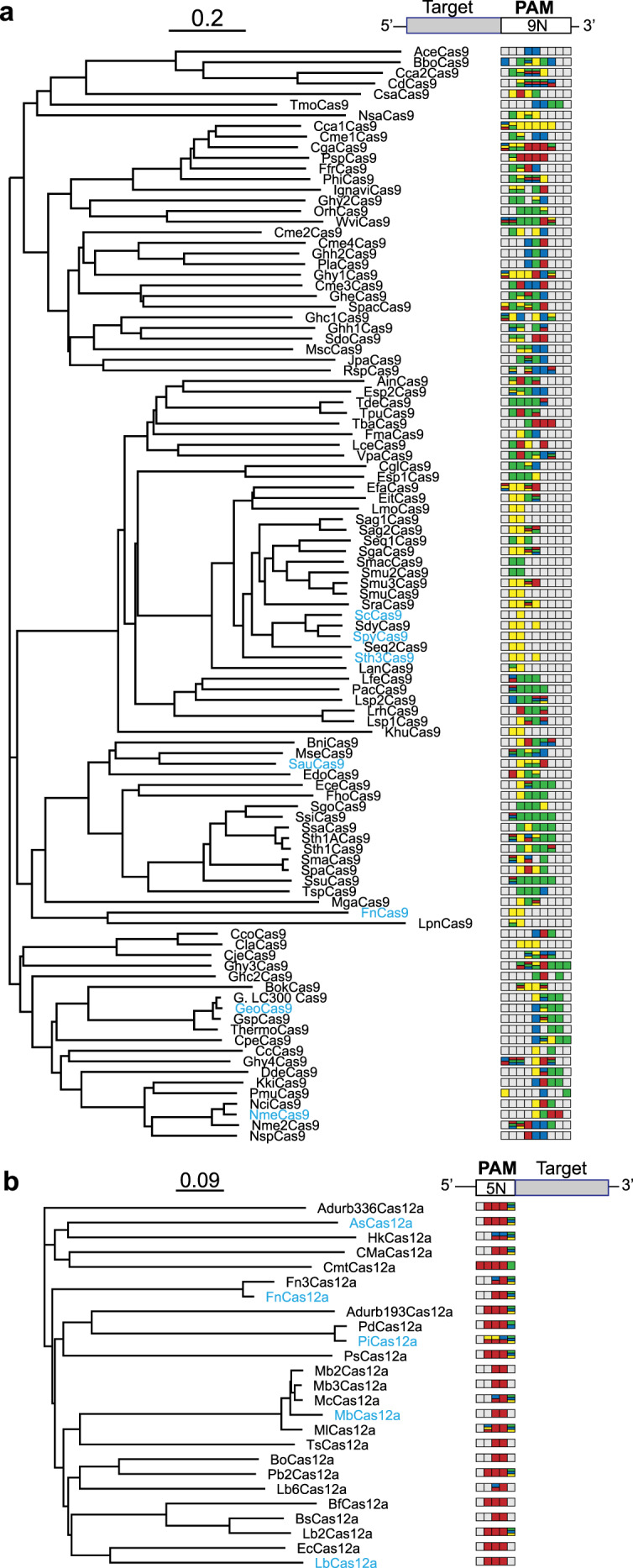
Phylogenetic relationship of PAM-characterized Cas9 and Cas12a nucleases found in nature. **a** Cas9 nucleases with characterized PAMs. **b** Cas12a nucleases with characterized PAMs. Phylogenetic trees with corresponding consensus PAMs are shown to the right. PAM-engineered variants are in light-blue text. The colors refer to each nucleotide: A = green, C = blue, G = yellow, and T = red. Stacked colors represent the recognition of at least two or three different nucleotides at the same position. Phylogenetic trees were generated using Geneious (Geneious Prime, version 2019.2.3, Biomatters Ltd.) based on the complete protein amino-acid sequence. Some of the nucleases recognized consensus sequences that slightly varied based on the PAM determination method or contained multiple motifs that were preferentially recognized, including those for CjeCas9^7^, ThermoCas9^8^, PdCas12a^33^, MbCas12a^50^, and Pb2Cas12a^50^. See Supplementary Data 1 for more details.

Beyond deeper characterization of a handful of Cas9 nucleases, efforts shifted to exploring the full diversity of Cas9 nucleases found in the natural world (Fig. 2a). To date, over 900 distinct Cas9 homologs have been identified in sequenced genomes and metagenomes^64^, and more homologs likely await discovery with further sequencing efforts. Exploring this expanded set has yielded a wide assortment of Cas9 nucleases with varying PAM profiles, protein sizes, and optimal activity temperature. One approach to prioritize within this massive set has been screening phylogenetically diverse Cas9 orthologs to identify the ones with unique PAM preferences. As one tour-de-force, Gasiunas et al. ^26^ screened over 70 Cas9 orthologs taken from ten distinct clades they identified. These extensive efforts uncovered an assortment of PAM profiles, including variants recognizing C-rich (RspCas9), T-rich (Cca1/PspCas9), and A-rich (OrhCas9) PAMs. Separately, amino-acid identity analyses comparing PID of SpyCas9 and other *Streptococci* Cas9 nucleases led to the identification of new orthologs with divergent PAM preferences^27,28^. Most notably, these efforts identified the *Streptococcus canis* Cas9 (ScCas9), which shares extensive homology to SpyCas9 outside of the PID, but recognizes an NNG PAM with a slight preference for an A at the second position^27^. This PAM profile represents one of the most relaxed profiles observed so far in nature. Furthermore, focusing on the two arginine residues in SpyCas9 that directly contact the PAM led to the identification of the Cas9 from *Streptococcus macacae* (SmacCas9) that has glutamines at the corresponding residues^28^. Chatterjee et al.^28^ hypothesized and experimentally demonstrated that SmacCas9 recognizes a consensus NAA PAM. When taken all together, the complete set of characterized Cas9 nucleases already covers ~65% of possible sequences when the consensus PAMs are aligned (Supplementary Fig. 1). However, the complete set covers ~92% of possible sequences if the PAMs can fall anywhere within a four-base window (Supplementary Fig. 1). PAM diversity thus has represented a common theme as other Cas9 nucleases beyond SpyCas9 have been characterized (Supplementary Data 1)^6–50^, potentially reflecting strong yet changing selective pressures on DNA-targeting requirements of CRISPR–Cas systems.

Despite the original and ongoing interest in Cas9, nature boasts an abundance of other CRISPR-associated single-effector nucleases that are still being discovered and harnessed as CRISPR technologies. Many of these nucleases even offer unique properties that open applications otherwise currently unavailable to Cas9. For example, Cas12a from Type V-A CRISPR–Cas systems generates a 5′ overhang as part of DNA cleavage instead of the blunt ends left by Cas9, processes its own gRNA from transcribed CRISPR arrays instead of requiring accessory factors similar to Cas9, and elicits collateral cleavage of single-stranded DNA upon target recognition that is not observed with Cas9^65^. Cas12a was first reported only five years ago^29,30^ but quickly became the most characterized class of nucleases second only to Cas9 (Supplementary Data 1). These efforts revealed that most of the characterized Cas12a nucleases recognize a T-rich PAM (Fig. 2b, Supplementary Data 1), e.g., TTTV (V = A, C, G) for the *Acidaminococcus* sp. Cas12a (AsCas12a)^29,31^. Less-preferred sequences have been shown to partially deviate from the consensus motif, such as AsCas12a accommodating a G at some positions^32^. However, a few Cas12a nucleases have emerged as outliers. The Cas12a from *Helcococcus kunzii* (HkCas12a) preferentially recognizes either two adjacent C’s at the second and third PAM positions as well as the standard PAM, resulting in a consensus of YYV^33,66^. Separately, the Cas12a from *Prevotella ihumii* (PiCas12a) exhibited the unique ability to recognize not only the TYV PAM but also guanine at the second, third, and/or fourth positions of the PAM (e.g., TTGC and GGCC)^33^. While representing only two examples, the PAM profiles for HkCas12a and PiCas12a suggest that further ortholog mining of Cas12a has the potential to identify additional and highly diverse PAMs.

Other subtypes of single-effector nucleases from Type V systems are still being discovered and hold potential for expanding PAM recognition^64^. To date, a handful of other Type V effectors has undergone PAM characterization (Supplementary Data 1), including Cas12b^34–36^, Cas12c^37^, Cas12d (formerly known as CasY)^38,39^, Cas12e (formerly known as CasX)^39^, Cas12f (previously known as Cas14 or from the subtype V-U3)^40^, Cas12j (or Cas12Φ) that forms the smallest known ribonucleoprotein complex^41^, and a Cas12k associated with a Tn7-like transposon^42^. More Type V subtypes have been recently discovered that remain to be characterized experimentally, leaving the potential for the discovery of new PAM recognition mechanisms as well as other CRISPR-based functions and technological breakthroughs.

Multi-subunit effector complexes from Type I and III systems have also been shown to exhibit properties distinct from any known single-effector nuclease^64^. For the abundant and phylogenetically diverse Type I systems, the Cascade complex responsible for target DNA binding generally recognizes flexible two- or three-base PAMs^51^, although PAMs have been determined for only a small number of these systems. Their ability to unidirectionally degrade DNA was recently exploited for extensive deletions in human cells^67^. In contrast, systems that search for RNA targets (i.e., from Type III and VI) do not recognize PAMs and instead evaluate the extent of complementarity between the flanking portions of the gRNA and target (see Box 1)^68,69^. The Cas13 single effectors from Type VI systems have been further exploited for programmable gene silencing equivalent to RNA interference^69^. The discovery and characterization of these nucleases expanded our understanding of PAM requirements, and it provides a foundation on which to obtain PAM-free nucleases for other CRISPR-based applications.

Efforts to delve into each subtype have operated under an overarching assumption: only phylogenetically distinct nucleases can recognize distinct PAM profiles. However, observations from the growing collection of characterized nucleases have begun to challenge this assumption. One important observation is that PAM profiles do not fully track with nuclease phylogeny (Fig. 2). Instead, recognized PAMs vary widely—and even between closely related homologs. Besides the previously discussed Streptococci Cas9’s, Edraki et al.^43^ identified related Cas9 orthologs in *N. meningitidis* strains with high sequence similarity everywhere, except for the PAM-interacting domain (PID). They found that representative members from different PID-aligned clusters recognized variations of the standard NNNNGATT consensus PAM for NmeCas9, including NNNNCAA, NNNNCAAA, and NNNNCCA. Separately, our group made similarly striking observations when investigating PiCas12a and the Cas12a from *Prevotella disiens* (PdCas12a)^33^. The two shares >95% amino-acid identity (including 96% shared identity in the PID) yet recognize distinct PAM profiles, with PdCas12a recognizing a more traditional TTYV consensus PAM. Mutating a subset of residues within the REC and WED domains in PiCas12a to match those in PdCas12a steered the PAM profile into a new territory, resulting in better recognition of G-containing PAMs than either parent nuclease. These insights establish the importance of comparing PID identity in Cas9’s and PI, REC1, and WED identity in Cas12a’s when mining orthologs in search of PAM diversity (Box 1). The insights also suggest that PAM recognition and other properties such as nuclease activity or gRNA binding could be under different selective pressures in nature. Overall, the known diversity of Cas nucleases supports PAM recognition as a flexible feature that can be altered with few mutations. This flexibility has been instrumental to the second means of obtaining Cas nucleases with more relaxed PAM recognition: protein engineering.

## Applying protein engineering to alter PAM recognition

In contrast to ortholog mining, protein engineering has proven to be a powerful means to alter and broaden PAM recognition starting from individual CRISPR nucleases. Protein engineering offers the means to steer proteins that evolved under biological pressures toward more technology-relevant applications, such as for genome editing or diagnostic detection. However, protein engineering poses multiple challenges. Each residue could be replaced with one of the 19 other amino acids, resulting in an astronomical number of combinations to screen for large portions of the protein. Individual mutations can also impact not just one but many properties of the protein, and mutations can impact these properties when introduced individually or in combinations^70^, requiring extensive downstream characterization. Accordingly, a range of approaches has been associated with protein engineering for altering PAM recognition, including random mutagenesis, structure-guided design, and chimera generation. We specifically focus on altering PAM recognition (Supplementary Data 2)^23,28,31,33,43–45,71–85^, although similar approaches have been applied to alter cleavage efficiency and the propensity for off-targeting^86^.

Initial efforts to alter PAM recognition began with SpyCas9, owing in part to its early adoption, robust activity, simple PAM, and the extensive knowledge base built around this nuclease. Kleinstiver et al.^23^ reported the first alteration of PAM recognition using SpyCas9 by combining random mutagenesis of the PID with a growth-based selection and subsequent counterselection. This approach yielded variants that shifted the consensus from NGG to NGA (VQR variant), NGAG (EQR variant), or NGCG (VRER variant)^23^. Furthermore, combining the most frequent mutations yielded the VRQR variant recognizing an NGA consensus PAM^73^. As most of these motifs were at least partially recognized by the WT SpyCas9, the end result was reshaping rather than recreating the PAM profile. The researchers also isolated a variant (D1135E) that exhibited reduced recognition of the less-preferred PAMs NGA and NAG, although a separate study showed that this variant still recognized other less-preferred PAMs like NNGG^25^.

**Fig. 3:**
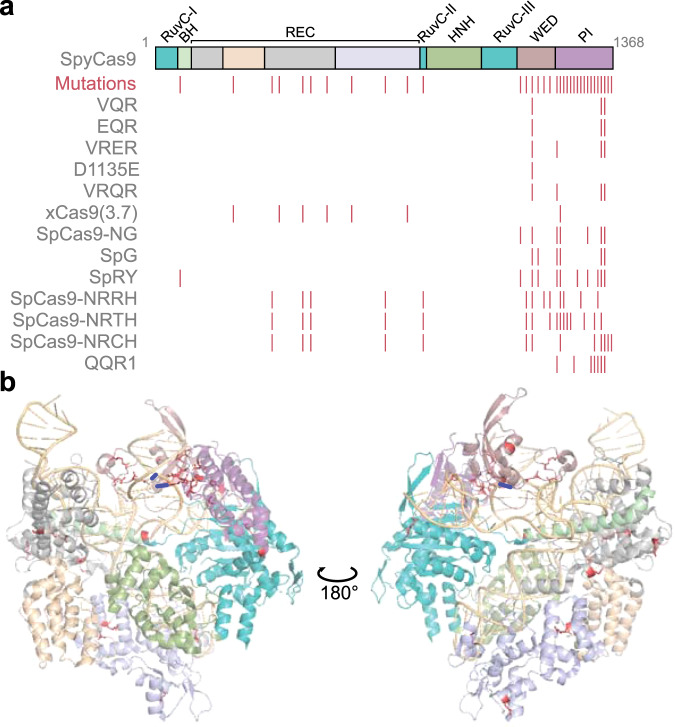
Mutations in the PAM-engineered variants of SpyCas9. **a** Domain architecture of SpyCas9. The location of the 34 mutated residues for all PAM-engineered variants (with the exception of chimeras) is indicated below the linear map. Each associated variant contains anywhere from 1 to 16 mutations. **b** Structure of SpyCas9 with mutated residues. Mutated residues are in salmon. The structural domains are colored according to the linear map in (**a**). The diguanine nucleotides in the PAM are shown as blue lines. Structural images were generated from PBD: 4UN3 using PyMOL (The PyMOL Molecular Graphics System, Version 2.4 Schrödinger, LLC).

The next set of engineering efforts aimed to broaden PAM recognition with a less-stringent motif, using the consensus NGG as a starting point. Hu et al.^74^ used a directed evolution approach called phage-assisted continuous evolution (PACE) to identify one variant dubbed xCas9(3.7) (or more simply xCas9)^87^. The researchers demonstrated that xCas9 could recognize NG with some preferences at the third PAM position along with GAW, CAA, and some NNG sequences. In addition, xCas9 was observed to exhibit reduced cleavage activity and less off-targeting^74,88,89^, paralleling some high-fidelity Cas9 nucleases that exhibit similar reductions. Correspondingly, the majority of the mutations in xCas9 were located within the REC domain, which is commonly mutated in high-fidelity Cas9 nucleases and undergoes a target-induced conformational change thought to precede DNA cleavage by the HNH and RuvC endonuclease domains^90^. Despite the reduced cleavage activity and dependence on the identity of the third PAM position, xCas9 represented a major advance on increasing PAM flexibility. As immediate competition to xCas9, Nishimashu et al. applied structure-guided design and mutant screening to develop their own relaxed variant of SpyCas9 called SpCas9-NG. The first mutated a key arginine (R1335) that directly contacts the second G in the NGG PAM, and they screened for mutations that introduce base-independent interactions to compensate for the lost PAM interaction. The resulting variant recognizes an NG consensus PAM^75^, with weaker recognition of NA PAMs. The resulting variant possessed seven mutations solely in the PID, one of which (E1219F) was also mutated in xCas9 (E1219V) (Fig. 3). Head-to-head comparisons between xCas9 and SpCas9-NG showed that the latter could more readily recognize sequences within the NG motif and exhibited greater indel formation and base editing in human cells^75^.

Although xCas9 and SpCas9-NG effectively required only a single G for the PAM, the most recent efforts further relaxed this requirement. Walton et al.^76^ set out to evolve a PAM-free SpyCas9 through further structure-guided mutagenesis of the VRQR variant. They began by sequentially screening mutations to key residues that impact PAM recognition. By screening an extensive list of mutant combinations, the researchers obtained two new variants: SpG and SpRY. SpG recognizes a consensus NG PAM and was shown to outperform xCas9 for all NGNN sequences and SpCas9-NG for two distinct NGNN sequences^76^. The SpRY variant recognizes a consensus NR (R = A or G) PAM, with less-preferred recognition of an RY (Y = C or T) PAM, thus demonstrating the most relaxed PAM preference to-date. As a result of the relaxed PAM preferences, the SpRY variant, in particular, demonstrated a higher tendency for off-targeting compared to SpyCas9—albeit based on a limited dataset^76^. However, high-fidelity mutations reduced off-target activity, as has been observed with other PAM-engineered variants^76–78^. Collectively, these variants represent the greatest progress to-date on engineering SpyCas9’s PAM preference, giving the sense that a PAM-free SpyCas9—a version that could recognize any sequence as a PAM—is almost within reach.

Aside from directing SpyCas9’s PAM preference toward a less-preferred PAM or relaxing the consensus motif, recent strides have been made to engineer nonnatural PAM preferences for SpyCas9. Miller et al.^79^ generated three SpyCas9 variants to attempt to guide the PAM preference toward NRRH, NRTH, and NRCH motifs (H = A, C, T), called SpCas9-NRRH, SpCas9-NRTH, and SpCas9-NCRH, respectively. Although the reported data indicate a more complicated PAM profile than the specified motifs, all three variants recognize PAM profiles that differ from the WT SpyCas9. There was also a bias for a G at the second PAM position and a clear preference for a T at the third PAM position for the NRTH variant (Supplementary Data 2). These variants were painstakingly generated using phage-assisted noncontinuous evolution, three separate PACE screens for each motif, followed by DNA shuffling and extensive characterization^79^. The resulting variants contained mutations primarily in the PID but also in the REC and HNH domains. Perhaps, for this reason, all three variants exhibited reduced off-targeting compared to SpyCas9^79^. In total, these three variants represent the first attempt to engineer SpyCas9 to recognize novel PAM profiles rather than a more relaxed consensus PAM.

In addition to the ongoing efforts to alter PAM recognition by SpyCas9, similar engineering approaches are also being applied to other Cas9 orthologs. For example, Hirano et al.^44^ relied on a crystal structure of the large *Francisella novicida* Cas9 (FnCas9) to relax PAM recognition from the consensus NGG to YG (Y = C, T). Separately, two groups^80,81^ relaxed PAM recognition by SauCas9 through random mutagenesis of the PID or structure-guided mutations (Supplementary Data 2). Finally, splicing divergent portions of the PID between otherwise similar homologs has allowed a distinct means of PAM engineering through the creation of protein chimeras. Chatterjee et al.^77^ compared the PID of ScCas9 to closely related orthologs, resulting in the identification of a lysine residue from *Streptococcus gordonii* and a positively charged loop from *Streptococcus anginosus* predicted to enhance nonspecific interactions with DNA. Splicing these two features into ScCas9 yielded Sc++, which recognized an NNG consensus PAM with little dependencies on the surrounding bases. In a separate study from the same group, the PID of *Streptococcus macacae* (SmacCas9), which was predicted to recognize an NAA PAM, was spliced into SpyCas9. The resulting chimera, dubbed SpyMac, recognized NAA despite otherwise resembling SpyCas9^28^. Using a similar approach, Ma et al.^78^ created chimeric versions of SauCas9 (cCas9) by replacing its PID with those from different related Cas9 homologs. These variants generally exhibited relaxed recognition at some PAM positions, although some recognition became more stringent at other sites. Similarly, other groups^43,45^ have made chimeras from closely related orthologs from *Neisseria* and *Geobacillus* to swap PAM profiles (Supplementary Data 2). The natural diversity of PAM preferences can therefore be exploited to meld engineering approaches and create variants that recognize new profiles. In total, the engineered SpyCas9 variants collectively cover ~56% of all possible sequences when the consensus PAMs are aligned and ~94% of the consensus PAMs can fall anywhere within a window of four bases. Furthermore, incorporating the NmeCas9 chimera recognizing an NNNNCC PAM raises this percentage for the four-base window to ~97%, covering the vast majority of potential sequences if some flexibility in the target location is acceptable (Supplementary Fig. 1).

PAM engineering is expanding beyond Cas9 to other CRISPR nucleases with unique properties. To date, multiple engineering efforts have altered the PAM profile of different Cas12a variants using some of the early approaches applied to SpyCas9. For example, Gao et al.^31^ altered PAM recognition by the widely used AsCas12a. Here, the researchers leveraged a crystal structure to identify and screen mutations in and around the PID, in turn identifying two variants (AsCas12a-RR and AsCas12a-RVR) that effectively shifted the consensus PAM from TTTV to TYCV and TATV, respectively. More recent work from Kleinstiver et al.^82^ applied targeted mutagenesis to AsCas12a based on its crystal structure. They identified an enhanced variant called enAsCas12a that exhibited a more relaxed PAM profile, although recognized PAM sequences did not conform to a single-consensus motif (Supplementary Data 2). Interestingly, AsCas12a-RVR and enAsCas12a shared two out of their three mutated residues and transferring these and other equivalent mutations to the Cas12a orthologs FnCas12a, LbCas12a (from *Lachnospiraceae* bacterium), and MbCas12a (from *Moraxella bovoculi*) resulted in similar alterations to the PAM profile^31,83,84^. Finally, a recent study from Liu et al.^85^ generated the first chimeric Cas12a by replacing two domains (WED-I and REC1) implicated in PAM recognition in the Cas12a ortholog MAD7 with that from the Cas12a from *Thiomicrospira* sp. (TsCas12a). The chimera exhibited more stringent PAM recognition, although it demonstrated the principle of creating Cas12a chimeras to alter PAM profiles. Overall, the groundwork is laid to alter PAM recognition by Cas12a and the many other recently discovered Cas nucleases distinct from Cas9.

## Anticipated trade-offs with a PAM-free nuclease

The field continues taking large strides toward a truly PAM-free nuclease. Engineering efforts applied to SpyCas9 have relaxed this nuclease’s PAM profile to roughly one of two bases at a single position—or 50% of possible DNA sequences. Following closely behind are efforts to engineer other Cas9 nucleases exhibiting distinct properties (e.g., smaller size and higher thermostability) as well as modified Cas12a nucleases. However, with PAM-free nucleases seemingly within reach, it is worth reflecting on what is gained and what is lost—and whether any change in course is warranted.

**Fig. 4:**
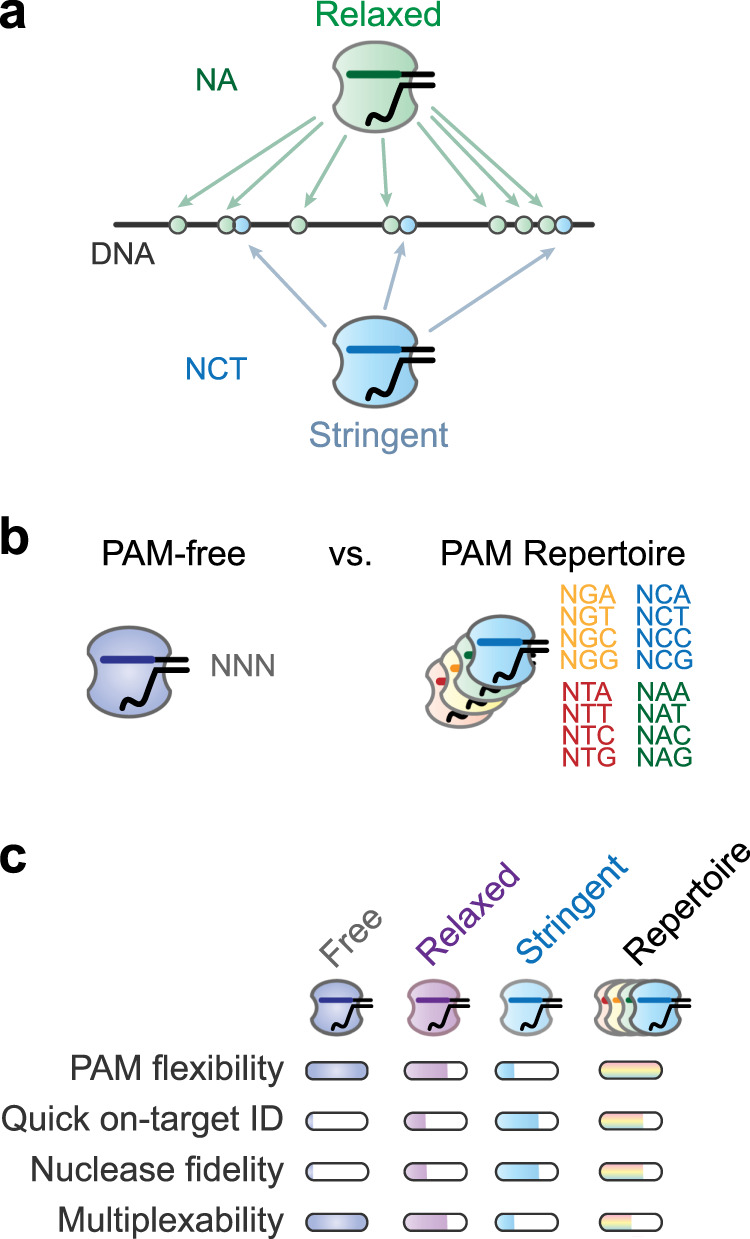
Implications of PAM engineering. **a** Comparing target accessibility for Cas nucleases with relaxed or stringent PAM requirements. **b** The PAM-free nuclease versus a repertoire of nucleases that collectively recognize every possible sequence. Here, the repertoire consists of four nucleases recognizing one letter at the second position. **c** Qualitative comparison of a PAM-free nuclease, a PAM-relaxed nuclease, a PAM-stringent nuclease, and the nuclease repertoire. The nucleases are compared using different metrics of targeting performance. The more a bar is filled, the greater our prediction the associated nuclease can perform under that metric. We consider nuclease fidelity as the ability of the nuclease to ignore nontarget sequences at least partially matching the guide sequence. We consider multiplex ability as how readily the nuclease can be implemented to target any set of genomic sequences.

The major upside of a PAM-free nuclease is clear: the ability to, in theory, target any sequence (Fig. 4). This flexibility would greatly simplify the selection of sites with high on-target but low off-target activity, generating predictable disruptive indels^91^, or placing the base-editing window directly over the target nucleotide. Any of these benefits would be further magnified when multiplexing because only one nuclease is necessary to simultaneously target any set of sequences. However, there are serious downsides worth considering (Fig. 4c). For gRNAs expressed from DNA constructs, self-targeting of this DNA would be immediate, unavoidable, and likely disastrous—highlighting the entire reason why CRISPR–Cas systems evolved PAMs (Box 1). In bacteria, self-targeting with a catalytically active nuclease would lead to the clearance of the gRNA-encoding plasmid or, for genomically integrated constructs, cell death^92^. In eukaryotes, self-targeting by a catalytically active nuclease would lead to indel formation within the guide, resulting in a modified guide sequence that can continue self-targeting until a defective gRNA is expressed. While this self-targeting strategy has been instrumental for lineage tracking^93^, it would quickly lead to inadvertent and potentially unpredictable targeting by the resulting progression of modified guides in other CRISPR-based applications. Even when using a catalytically dead nuclease for CRISPR interference or activation^1,94^, a gRNA would block its own transcription.

As a separate downside, a nuclease with no PAM requirements would also be expected to interrogate every sequence in the genome. Such thoroughness in target scanning could present two issues: extended timescales for the nuclease to find its target and an increased propensity for off-targeting (Fig. 4b). The extended timescale would arise from the need to interrogate every possible PAM-flanked site, as evidenced by the increased lifetime of Cas9 on DNA with higher PAM densities in vitro^95^. The end effect would be reduced editing efficiency, even if binding and cleavage rates match that of a standard nuclease. Separately, interrogating every possible site would give the nuclease ample opportunities to cleave potential off-target sites. Accordingly, there is some evidence that the engineered variants SpG, SpRY, and enAsCas12a recognizing relaxed PAMs exhibited increased off-targeting compared to their parent proteins^76,82^. Fortunately, adding mutations that reduce mismatch tolerance could counteract this effect and even improve the frequency of on-target editing, such as was done to generate high-fidelity versions of SpRY, Sc++, and enAsCas12a^76,77,82^. Even without off-target cleavage, transient occupancy of nonspecific sites across the genome could instigate genomic instability and cytotoxicity, as observed when overexpressing the catalytically dead SpyCas9 in *Escherichia coli*^96^. Finally, introducing a disruptive mutation to the PAM is generally the most dependable means of creating a defined edit no longer recognized by the guide, particularly for single-base edits. While this strategy would no longer be applicable for PAM-free nucleases, relying on a high-fidelity version of the nuclease and disruptive mutations in the target could achieve the same outcome. Therefore, we posit that a PAM-free nuclease may not be universally applicable for every CRISPR technology and instead comes with real trade-offs that could compromise some applications.

## Future perspectives and outlook

Given the potential drawbacks of a PAM-free nuclease, how should the field proceed? First, while engineered SpyCas9 nucleases are almost PAM-free, the abundance of other Cas9, Cas12a, and the remaining Cas nucleases have ample room for relaxing PAM recognition before approaching PAM-free status. To accelerate developments with these nucleases, a combination of ortholog mining and PAM engineering offers a fruitful and expedient path, such as that followed to create Sc++^77^. Within ortholog mining, the set of characterized Cas9 nucleases indicates that exceptional PAM diversity exists within nature and remains to be fully uncovered. Future work could delve into established but poorly characterized CRISPR–Cas types, such as Type I and V systems, that have been recently repurposed for different CRISPR technologies^42,67,97–99^. Doing so could present convenient starting points for further engineering, such as Type V–C nucleases that recognize PAMs with as little as a single base^37^. Solving the structure of nucleases naturally recognizing only one nucleotide could reveal distinct modes of PAM recognition that could motivate future structure-guided engineering of these nucleases. The generation of chimeras from two similar homologs also highlights the benefit of splicing existing nucleases, although structure-based approaches such as SCHEMA could more effectively guide splicing of nuclease domains and mediate the large-scale screening of chimeras^100^. Incorporating screening approaches that alternate between protein stability and function could also open regions of sequence space otherwise considered inaccessible through single-point mutations^101^. Finally, through these combined efforts, we envision the accrued datasets laying the foundation for computer-aided design of nucleases with defined PAM profiles, whether through molecular modeling or machine learning.

Cas nucleases exhibit a wide range of properties beyond PAM recognition important for different applications. These properties include size, protein folding, gRNA recognition and processing, binding and cleavage rates, propensity for off-targeting, temperature dependence, host immune response^102,103^, and performance in different cellular contexts. Current efforts to alter the PAM profile have endeavored to determine not only the full profile through a range of high-throughput techniques^51^ but also to investigate on-target efficiency and off-targeting. However, these evaluations have not always been fully conducted, and the other properties of CRISPR nucleases are often neglected. The field of directed evolution has a common phrase^104^: you get what you screen for. In the case of Cas nucleases, neglecting the various properties as part of any screen can allow these properties to stray and likely become less optimal. For example, the generation of the engineered variants SpCas9-NRRH, SpCas9-NRTH, and SpCas9-NRCH relied on binding activity by a catalytically dead Cas9, and the early round variants exhibited reduced or abolished cleavage activity^79^. In the future, incorporating assays for these other properties could become a benchmark for introducing engineered nucleases, and these assays could eventually be incorporated into high-throughput screens that become part of the testing pipeline. In turn, future engineering efforts could alter the entire length of the nuclease, generating versions bearing little resemblance to their natural counterpart.

As a final point, we put forward an alternative that the field could pursue besides PAM-free nucleases: a nuclease repertoire (Fig. 4b). Here, each nuclease retains recognition of a defined PAM, whether the PAM is a single base (e.g., NG) or a series of bases (e.g., NAAA). A collection of these nucleases could be explicitly assembled to cover all possible sequences, thereby achieving a collective PAM-free status. Because each nuclease would retain PAM recognition, it would avoid some of the drawbacks discussed above when PAMs are no longer a requirement. A researcher would then select from this repertoire based on the desired target, using the flanking sequence to determine which nuclease should be employed. This design approach would represent the converse of the current practice in which the target is selected based on the available nuclease. Clearly, some researchers are thinking along these lines, based on claims of the percentage of possible sequences covered by a set of engineered variants^74–76,79^. However, achieving a true repertoire would require a different approach. For one, it would require settling on the right balance between PAM specificity and repertoire size. For another, it would require prioritizing efforts to complement existing nucleases and ensuring that, aside from PAM recognition, the nucleases behave as similarly as possible. For example, further engineering efforts could focus on the few C/T-containing sequences not extensively covered by engineered SpyCas9 variants (Supplementary Fig. 1), such as by incorporating structural insights from Cas9 nucleases recognizing T-rich PAMs or the NmeCas9 chimeras that recognize an NNNNCC PAM (Supplementary Data 1 and 2). For multiplexing applications, priorities could be centered around creating variants that recognize not only different PAMs but also different gRNA scaffolds^46,47^. Expressing multiple nucleases would be challenging for many applications, although efforts to express domains as split proteins or relying on alternative splicing could reduce the DNA footprint of the resulting constructs. Overall, developing the nuclease repertoire could be even more within reach and bring us to a point where any sequence can be the target of CRISPR technologies.

## Supplementary information

Supplementary Information

Description of Additional Supplementary Files

Supplementary Data 1

Supplementary Data 2
