# Supplementary material for: CRISPR technologies and the search for the PAM-free nuclease
Source: Nat Commun. 2021 Jan 22;12:555. doi: 10.1038/s41467-020-20633-y (PMC7822910; doi:10.1038/s41467-020-20633-y)
Supplement: Supplementary file 1 — Supplementary Information [file 41467_2020_20633_MOESM1_ESM.pdf]

CRISPR technologies and the search for the PAM-free nuclease

Collias and Beisel

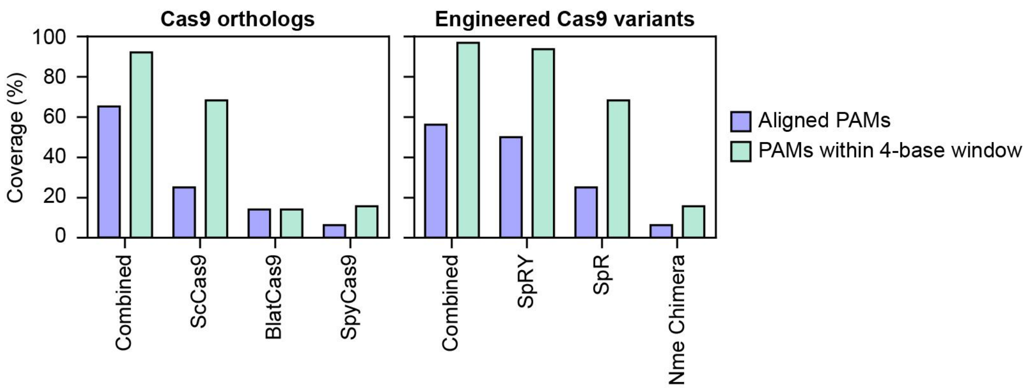

Supplementary Figure 1. The PAM sequence space covered by natural Cas9 orthologs and engineered Cas9 variants. The sequence coverage was determined by calculating the fraction of sequences a Cas9 ortholog or variant could theoretically target when the consensus PAMs are aligned (lavender) or fall anywhere within a 4-base window (aqua). The calculations are based on a 4N library and ignoring less-preferred PAMs for each nuclease (e.g. NAG for SpyCas9). The combined sequence space coverage was calculated by combining all PAM profiles of the natural Cas9 orthologs (left) or engineered variants (right).
