# Supplementary material for: CRISPR technologies and the search for the PAM-free nuclease
Source: Nat Commun. 2021 Jan 22;12:555. doi: 10.1038/s41467-020-20633-y (PMC7822910; doi:10.1038/s41467-020-20633-y)
Supplement: Supplementary file 2 — Description of Additional Supplementary Files [file 41467_2020_20633_MOESM2_ESM.pdf]

Title: Supplementary Data 1.
Description: Complete list of Type II and Type V Cas orthologs with characterized PAMs. Information regarding % identity to SpyCas9 or FnCas12a, the PAM determination method, the validation method, and whether a crystal structure is available are also included.

Title: Supplementary Data 2.
Description: List of engineered Type II and Type V Cas nuclease variants. Information regarding the PAM determination method and off-target analysis are also included
